# Combined effects of functionally-oriented exercise regimens and nutritional supplementation on both the institutionalised and free-living frail elderly (double-blind, randomised clinical trial)

**DOI:** 10.1186/1471-2458-9-39

**Published:** 2009-01-28

**Authors:** Marek Zak, Christian Swine, Tomasz Grodzicki

**Affiliations:** 1 Chair of Clinical Rehabilitation, University School of Physical Education, Krakow, Poland; 2 Department of Geriatrics, University Hospital Mont-Godinne, Catholic University of Louvain, Louvain, Belgium; 3 Chair of Internal Medicine and Gerontology, Jagiellonian University School of Medicine, Krakow, Poland

## Abstract

**Background:**

Consistently swelling proportion of the frail elderly within a modern society challenges the overstrained public health sector to provide both adequate medical care and comprehensive assistance in their multiple functional deficits of daily living. Easy-to-apply and task-specific ways of addressing this issue are being sought out, with a view to proposing systemic solutions for nationwide application.

**Methods:**

The present randomised, double-blind, placebo-controlled, 7-week clinical trial aimed to determine whether specifically structured, intensive exercise regimens, combined with nutritional supplementation, might improve and help sustain individual muscle strength and mobility, and possibly enhance individual functional capabilities in an on-going quest for active prevention of care-dependency. Ninety-one frail elderly (F 71 M 20; mean age 79 years) were recruited from both nursing home residents and community dwellers and randomly split into four groups: Group I – progressive resistance exercises (PRE) + functionally-oriented exercises (FOE) + nutritional supplementation (NS), Group II – PRE + FOE + placebo, Group III – standard exercises (SE) + FOE + NS, Group IV – SE + FOE + placebo. Each group pursued a 45 min. exercise session 5 times weekly. The subjects' strength with regard to four muscle groups, i.e. hip and knee extensors and flexons, was assessed at 80% (1 RM) weekly, whereas their balance and mobility at baseline and at the end of the study.

**Results:**

The study was completed by 80 subjects. Despite its relatively short duration significant differences in muscle strength were noted both in Group I and Group II (p = 0.01; p = 0.04; respectively), although this did not translate directly into perceptible improvement in individual mobility. Notable improvements in individual mobility were reported in Group III and Group IV (p = 0.002), although without positive impact on individual muscle strength.

**Conclusion:**

Comprehensively structured, high-intensity regimen made up of diverse exercise types, i.e. functionally-oriented, progressive resistance and standard ones, preferably if combined with nutritional supplementation in adequate volume, demonstrates clear potential for appreciably improving overall functional status in the frail elderly in terms of individual walking capacity and muscle strength.

**Trial registration:**

Central Register of Clinical Trials, Poland – CEBK180/2000.

## Background

Human life expectancy has steadily been increasing over the past several decades, much aided by momentous advances in medical science. Modern society is nowadays invariably faced with the daunting challenge of having to care for its consistently swelling proportion of the frail elderly. Their individual expectations in overall quality of life by no means diminish with advancing age, usually remaining at odds with their much impaired functional capabilities and therefore causing much grief and frustration to themselves and their carers alike [1-5].

A vast majority of the elderly are widely acknowledged to experience significant decline in their overall functional capacity, primarily originating from advancing age, concomitant chronic illnesses and frequently inadequate nutritional intake. This consequently makes them heavily dependent on others for their daily functional routine, especially with regard to mobility, let alone running a relatively high risk of sustaining accidental falls, serious fractures, recurrent hospitalisations and ultimately an admission into a nursing facility [6-9]. Such dependency in turn tangibly erodes overall quality of their lives, as well as the all-important sense of self-appreciation.

Functional deficiency experienced by the elderly, as routinely encountered by physicians, is in fact expected to take up a significant proportion of their medical practice. Whereas modern science and technology should definitely seek out innovative and more effective ways of facilitating adequate medical treatment to the seniors, it should also focus on finding effective ways of combating their growing dependency on outside assistance through introducing feasible and easy-to-apply measures. Those should primarily be designed to interact effectively with their predominantly sedentary lifestyles, also with a view to persuading them that even a little effort on their part, especially when structured as a specifically targeted intervention and regularly pursued, may in fact make a tremendous and lasting difference to overall quality of their lives [10-13].

Since an overwhelming body of evidence suggests that a loss of muscle strength and function observed with advancing age is reversible even in the frail elderly, it might reasonably be assumed that such a reversal might well be facilitated by a comprehensively structured solution involving task-specific exercise interventions, possibly combined with adequate nutritional supplementation [2,3,14]. Provided its overall effectiveness has been proved beyond reasonable doubt, it might consequently become instrumental in appreciably aiding improvement of individual activity level and significantly enhancing individual functional capacity. Furthermore, it might by the same token dramatically reduce the growing dependence of the elderly on outside assistance in their activities of daily living, whilst effectively promoting greater self-reliance, and – last but not least – significantly boosting up their self-confidence.

These key considerations prompted the authors to focus primarily on the applied aspect of the study, seeking out to design a feasible intervention routine, ostensibly with a view to having it subsequently utilised as a core part of a nationwide public health project aimed at helping the frail elderly cope effectively with the activities of daily living. The need for such a comprehensive, nationwide project to be put in place is already paramount, its urgency underpinned by the fact that the generation of baby-boomers is now approaching a critical milestone – their retirement age.

## Methods

### Study design

The present study aimed therefore to verify the working hypothesis that a comprehensive, functionally-oriented exercise regimen, when pursued in conjunction with adequate nutritional supplementation, might appreciably improve individual activity level and significantly enhance individual functional capacity (i.e. muscle strength, walking capacity and balance) in the frail elderly.

It was designed as a randomised, double-blind, placebo-controlled, 7-week clinical trial in which the subjects, randomly split into four intervention groups, were assigned to participate in two discrete types of functionally-oriented exercise (FOE) regimens incorporating elements of balance and multi-sensory training: either in the structured standard exercises (SE) or the structured progressive resistance exercises (PRE), both regimens pursued in conjunction with either multi-nutrient or placebo supplementation.

Double-blinding with regard to nutritional supplementation was assured as neither the study subjects, nor indeed the nursing staff had any prior knowledge of the actual composition of the identically packaged drink. With respect to the exercise regimen, each set of exercises was supervised by a separate group of outsourced, free-lance physiotherapists who had no knowledge of the actual nature of the complementary set of exercises, nor did they come into personal contact with each other in any of the settings, so no inferences could possibly have been made by them as to the true purpose of the two combined regimens.

Throughout the course of the study all pertinent data were collected either by the nursing staff, or the physiotherapists, who all had successfully completed the Good Clinical Practice training scheme, and then duly collated by the Project Leader.

The present study protocol was duly endorsed by an Independent Ethics Committee (The M. Sklodowska – Curie Centre of Oncology, Krakow) as fully compliant with the 1964 Helsinki Declaration, as well as a written informed consent was secured from each study participant.

### Study population

Volunteers were recruited from a nursing home facility (42), as well as from the former patients of the University Clinic Geriatric Ward (49), subsequently becoming the free-living study subjects (i.e. community dwellers).

**Figure 1:**
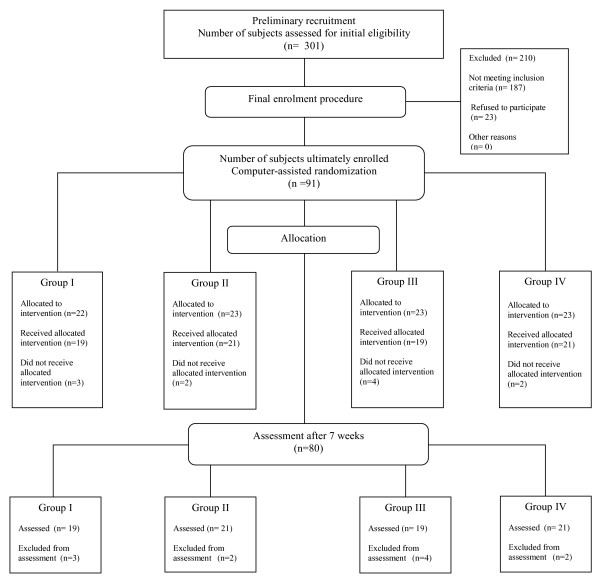
**Flow of participants through the trial**.

Out of 301 original volunteers 91 subjects (mean age 79 ± 7.6) were ultimately enrolled into the study (Figure 1) as fully compliant with the following inclusion criteria:

1. Age ranging 60 – 95 years

2. Body weight (being overweight within a 20% range)

3. Body Mass Index (BMI) > 19

4. Individual balance, as evidenced by > 21 score on the Berg Balance Scale (BBS)

5. Score of > 20 on the Mini Mental State Examination (MMSE)

6. Lack of any counter-indications on medical grounds (i.e. no currently administered medication interfering with individual balance and overall movement co-ordination)

The following exclusion criteria were also stringently applied:

1. Cancerous disease in the stage of advancement rendering a subject's participation in the trial non-feasible

2. Prior surgical treatment of the abdominal area within three months prior to the commencement of the study

3. Acute gastric tract disorders

4. Acute pancreatitis or diabetes

5. Any recently sustained fractures (i.e. within the year immediately preceding the commencement of the study)

6. Any past cerebral incidents whose lasting functional after-effects would make the pursuit of exercise regimens non-feasible (e.g. hemiplegia accounting for a stiff knee joint)

Out of 91 subjects ultimately enrolled into the study, 11 dropped out within the first fortnight (i.e. 10 on their own accord, and 1 due to a serious adverse event, although this was neither due to the supplementary nutrition, nor indeed to the nature of the actually pursued exercise regimen), thus reducing down to 80 the overall number of participants who successfully completed the study (i.e. 38 nursing home residents and 42 community-dwellers).

### Baseline characteristics

Medical, functional and overall mental/cognitive status of the study subjects was assessed and documented using the following standard evaluative procedures: select blood tests, ECG, lung X-ray, the Katz Index Activities of Daily Living (ADL) [15], the Lawton/Brody Instrumental Activities of Daily Living (IADL) [16] and Mini-Mental State Examination (MMSE) [17,18]. Assessment for medication (i.e. the number of medications routinely used by the respective subjects, although without any discrimination into specific types), Mini Nutritional Assessment (MNA) [19], Body Mass Index (BMI) was also completed. Overall, there were no statistically significant differences between the respective study groups prior to the commencement of the intervention protocol. Baseline characteristics are listed comprehensively in Table 1.

**Table 1:** Baseline characteristics of the study population

**Characteristics**	**Group I (n = 19)** PRE+FOE+nutritional supplement	**Group II (n = 21)** PRE+FOE + placebo	**Group III (n = 19)** SE+FOE+nutritional supplement	**Group IV (n = 21)** SE+FOE + placebo
Age, mean ± SD	78.1 ± 7.6	79.2 ± 9.2	78.3 ± 6.8	81.1 ± 6.4
Female sex (%)	84	81	74	81
Height (cm) mean ± SD	159 ± 5.3	160 ± 8.7	159 ± 7.5	158 ± 9.3
Weight (kg) mean ± SD	62.2 ± 13.2	63.4 ± 12.1	63.8 ± 12.3	63.9 ± 11.2
Systolic blood pressure (mmHg)	138 ± 9.9	129 ± 18.1	136 ± 17.5	140 ± 18.1
Diastolic blood pressure (mmHg)	85 ± 7.9	80 ± 9.5	81 ± 7.0	82 ± 9.5
Heart rate mean ± SD	77 ± 9.3	75 ± 6.5	76 ± 4.8	76 ± 10.5
**Diagnoses/medications**				
Cardiovascular diseases (%)	79	67	74	76
Pulmonary disorders (%)	53	48	58	52
Locomotive disorders (%)	63	62	68	67
Neurological disorders (%)	37	38	32	43
Urinary system diseases (%)	21	19	26	24
Visual deficits (%)	47	43	42	38
Regular medications Mean ± SD	8.3 ± 5.6	9.1 ± 4.4	8.6 ± 5.1	9.4 ± 4.6
**Key inclusion criteria**				
Berg Balance Scale (0–56) mean ± SD	45 ± 9.5	47 ± 8.2	46 ± 8.3	44 ± 9.2
MMSE (0–30) mean ± SD	23.8 ± 3.7	23.5 ± 4.5	24.7 ± 5.9	23.7 ± 4.0
**Nutritional status**				
BMI (kg/m^2^) mean ± SD	24.7 ± 0.8	24.3 ± 0.6	25.2 ± 0.7	25.2 ± 0.6
MNA score (0–30) mean ± SD	22.1 ± 2.6	23.1 ± 2.5	23.3 ± 2.2	22.4 ± 2.2
Na mmol/l mean ± SD	142.8 ± 5.1	143.8 ± 1.9	144.1 ± 2.7	143 ± 2.7
K mmol/l mean ± SD	4.1 ± 0.4	4.2 ± 0.5	4.1 ± 0.4	4.3 ± 0.5
Ca mmol/l mean ± SD	1.8 ± 0.6	2.1 ± 0.7	1.8 ± 0.7	1.8 ± 0.7
Glucose mmol/l mean ± SD	4.9 ± 0.57	4.8 ± 0.57	4.8 ± 0.54	4.9 ± 0.86
Total cholesterol mol/l mean ± SD	5.09 ± 1.57	6.35 ± 1.04	5.54 ± 1.07	6.07 ± 1.02
LDL mmol/l mean ± SD	3.45 ± 1.2	4.25 ± 1.0	3.92 ± 0.9	4.18 ± 0.9
HDL mmol/l mean ± SD	1.44 ± 0.4	1.65 ± 0.6	1.45 ± 0.4	1.39 ± 0.5
**Functional capability**				
ADL (0–6) mean ± SD	5.4 ± 0.8	5.5 ± 0.8	5.4 ± 1.0	4.9 ± 1.1
IADL (0–27) mean ± SD	20.8 ± 5.4	21.4 ± 5.5	20.7 ± 4.8	20.1 ± 4.7
Tinetti Total score (0–28) Mean ± SD	22.3 ± 5.3	23.8 ± 4.1	22.5 ± 5.6	20.9 ± 4.8
Tinetti Balance score (0–16) mean ± SD	12.5 ± 3.1	13.6 ± 2.2	13.1 ± 3.5	11.0 ± 3.0
Tinetti Gait score (0–12) Mean ± SD	9.7 ± 3.0	10.2 ± 2.2	9.4 ± 2.5	9.9 ± 2.6
6 MW test (m.) mean ± SD	267 ± 129	222 ± 103	262 ± 106	228 ± 107
Use walking aids (%)	42	48	47	43
**Muscle strength (N/m.)**				
**Right leg**				
Knee flexion mean ± SD	18.1 ± 7.0	18.5 ± 6.4	17.7 ± 5.3	18.6 ± 6.3
Knee extension mean ± SD	33 ± 12.7	36 ± 9.9	34.5 ± 13.4	33.5 ± 10.9
Hip flexion mean ± SD	27.1 ± 12.6	29.9 ± 13.2	29.6 ± 9.6	28.5 ± 9.6
Hip extension mean ± SD	21.2 ± 7.3	21.3 ± 8.5	20.2 ± 8.5	20.3 ± 8.2
**Left leg**				
Knee flexion mean ± SD	18.3 ± 6.5	17.9 ± 5.8	17.7 ± 5.2	18.9 ± 6.7
Knee extension mean ± SD	33.6 ± 13.6	36.8 ± 13.0	33.1 ± 12.2	34.9 ± 13.8
Hip flexion mean ± SD	29.8 ± 10.5	31.9 ± 11.8	31.1 ± 13.1	30.5 ± 10.6
Hip extension mean ± SD	22.4 ± 7.3	22 ± 9.9	21.6 ± 5.9	22.1 ± 7.4

PRE – Progressive resistance exercises, FOE – Functionally-oriented exercises, SE – Standard exercises, SD – standard deviation, MMSE – Mini-Mental State Examination, BMI – Body Mass Index, MNA – Mini Nutritional Assessment, LDL – Low-density lipoprotein cholesterol, HDL – High-density lipoprotein cholesterol, ADL – Activities of Daily Living, IADL – Instrumental Activities of Daily Living, 6 MW – Six-Minute Walk test

### Randomisation of study population

Following the completion of the recruitment stage the participants were randomly assigned (through computer-assisted randomisation) to either the control, or intervention group after the baseline testing. Allocation of study subjects into the respective groups envisaged equal distribution of both the nursing home residents and former geriatric ward patients. The assessors were duly blinded to the group allocations in respect of all outcome measures.

### Overall assessment of physical function

In order to be admitted into the testing procedure all potential participants were required to pass successfully the Berg Balance Scale test, i.e. the required score > 21 points [20,21], which was specifically meant to screen out any subjects with substantially impaired mobility function, as well as all those prone to incidental falls.

The entire study population was then assessed in terms of individual functional performance capabilities using two standard test procedures: Six-Minute Walk test (6 MW) [22,23] and Tinetti's Performance Oriented Mobility Assessment test, as subsequently modified (POMA) [24,25].

### Muscle strength assessment

The measurements were taken with the aid of HOGGAN MicroFET2 dynamometer (Hoggan Health Industries, Draper, Utah) [26-28], using complex strain gauge elements located within the transducer to achieve high degree of accuracy. Depending on the respective read-outs, the subjects were individually allocated a particular type of the standardised resistive exercise band (Thera-Band^®^), in full consideration of their individual physical capacity, therefore ensuring truly progressive character of the training regimen.

Each subject was allowed two or three practice trials to familiarize himself with the actual procedure. Three formal measurements of a subject's muscle strength were then taken in four different lower limb positions and duly recorded (allowing for 1-minute rest periods between the respective trials), whereas the best trial was ultimately accepted as the final result.

The measurements consequently allowed to determine 80% of the muscle strength within each specific muscle group (i.e. hip and knee extensors and flexons), selected in view of their significant impact upon general functional activities. This value was then verified by one repetition maximum (1-RM), i.e. the maximal stretch of a rubber resistance band at one time only, as per interim (weekly) measurements in order to adjust the individualized training load accordingly.

The subjects' muscle strength was assessed twice, i.e. prior to the commencement of the PRE regimen and after its conclusion. All measurements were carried out by the same physiotherapist to ensure that there were no idiosyncratic differences in the actual application of the procedure. Full compliance with the GCP Guidelines was assured throughout.

### Mini Nutritional Assessment (MNA)

This assessment procedure was applied prior to the actual commencement of the study protocol, aiming to provide the attending physician with the structured information in the following categories: standard anthropometric measurements, inclusive of BMI (to screen out any possible nutritional deficit), global evaluation and dietetic assessment, expressed by a maximum score of 30 points.

### Assessment of nutritional intake

Initially, the habitual nutritional intake of every study subject was assessed by a full-time clinical dietician who charted the caloric value of an entire daily food intake of each patient for 7 days prior to the commencement of the study. The subjects were instructed by the dietician to complete an estimated record of all foods consumed during each day (including weekend) in as much detail as possible. The information supplied voluntarily by the subjects, as well as pertinent dietary data collected by the dietician off the actual nursing home menu cards, was then meticulously recorded in the standardised Case Report Forms (CRFs).

The same procedure was then extended over the entire length of the study, so as to facilitate strict monitoring of overall energy intake in full consideration of the nutritional supplementation. All records were duly coded and subsequently analysed for nutrient composition by the same person, using the MS EXCEL-based computer application, specifically designed for the purpose by the resident IT engineers.

### Intervention procedures

#### Exercise regimens

All exercise interventions were comprehensively structured by the authors, primarily with the aim of restoring the already impaired functional capabilities in the frail elderly through helping them build up overall fitness, with particular emphasis on individual balance, mobility and muscle strength.

Since all exercises are actually designed with a view to making active use of simple and easy to obtain props, i.e. pedal exercisers, ball cushions, standardised elastic resistance bands, household furniture, they are easily adaptable to different environments, as well as seem to hold much more appeal to the subjects than the highly sophisticated training equipment, frequently known to cause apprehension or even high anxiety [29,30].

The principal idea consisted in selecting such props that could easily be applied in the way effectively interacting with the subjects' predominantly sedentary lifestyle. For instance, the subjects were found to be quite at ease when balancing on a ball cushion on top of the chair seat, or pedalling away at the exerciser, whilst watching their favourite TV shows. This way the subjects did not have to divert much from their daily routines, merely having them slightly modified to accommodate actual application of the props. The obvious functional advantage of establishing such an easy-going exercise routine consisted in the fact that it effectively aided activation of cardiovascular function, stimulation of walking capacity and overall enhancement of individual balance. Finally, these exercises may be individually pursued without any supervision, once the actual routine has been established by a licensed physiotherapist.

#### Structured exercise sessions

Each exercise training session was designed to last 45 minutes and the regimen was to be pursued for 5 days a week (i.e. weekends were exercise-free for administrative reasons), which in the view of the present authors might reasonably be construed as a high-intensity activity. Each 45 min. session was further broken down into the following components: 5 min. general warm-up exercises of the upper and lower limbs and the trunk (initially in an recumbent position, and in a sitting down position on a chair afterwards), to be followed by ca. 20 min. of functionally-oriented exercises (FOE), to be then followed by ca. 20 min. long standard exercises (SE), or progressive resistance exercises (PRE), depending on a specific group allocation. Every session was then rounded off with a series of simple breathing and relaxation exercises. All exercise interventions were pursued at the subjects' actual place of residence and individually supervised by a physiotherapist to ensure full (100%) compliance with the study regimen (Table 2).

**Table 2:** The core structure of the study protocol

	**Exercise regimens pursued for 7 weeks, 5 times a week (once daily)**
**Group I**	Progressive resistance exercises (PRE) + Functionally-oriented exercises (FOE) + nutritional supplementation

**Group II**	Progressive resistance exercises (PRE) + Functionally-oriented exercises (FOE) + placebo supplementation

**Group III**	Standard exercises (SE) + Functionally-oriented exercises (FOE) + nutritional supplementation

**Group IV**	Standard exercises (SE) + Functionally-oriented exercises (FOE) + placebo supplementation

Functionally-oriented exercises (FOE) were allocated to all study groups specifically with a view to generally enhancing their respective functional capabilities prior to the actual commencement of the other two exercise regimens (SE and PRE) designed as the ultimate differentiation factor. As opposed to a diversity of other types of exercises generally recommended to the frail elderly by physiotherapists, the functionally-oriented ones are commonly acknowledged to be universally well tolerated, versatile and offer a multitude of tangibly beneficial effects, e.g. stimulate individual balance (especially when already impaired), promote safe postural shifts and enhance individual walking capabilities. Such a complex "warm-up" routine was also meant to have the subjects better prepared to face up to the much more challenging "power" exercises, as well as was expected to put all participants on the same functional platform, as it were, so that subsequent results could consequently lend themselves to more objective assessment in terms of their actual statistical relevance.

These exercises consisted of a series of 3 exercises designed to stabilise the subject's balance when rising from a standardised chair to a fully upright position. This was then followed by a series of multi-sensory exercises with the aid of a ball cushion, so as to give the subject an impression of an uneven, as well as an unstable surface on which he is then required to perform. All exercises were carried out in both sitting and fully upright position [31].

Standard exercises (SE) consisted of a series of 10 simple exercises (implemented in an upright sitting position on a standardised chair), followed by another series pursued on a pedal exerciser, designed to activate the lower limbs (duration: ca.10 min. with three 30 sec. intervals).

Progressive resistance exercises (PRE), meant to be pursued after prior completion of the warm-up set, were developed as the four series of proper resistance exercises pursued with the aid of a standardised elastic resistive exercise band [32,33]. Each series consisted of 3 × 10 stretches of a band per each discrete muscle group (Cf. Tables 3 and 4), spaced out with 1 min. long rest periods, designed specifically for restoring functionality of the lower limbs, in order to compensate for any lack of movement through stretching and strengthening the specific muscle groups, all with a view to improving overall muscle strength.

**Table 3:** Muscle strength – changes in outcome measures between groups: baseline values vs. scores after 7 weeks.

**Muscle strength (N/m.)**	**Group I (n = 19)** PRE+FOE+ nutritional supplement	**Group II (n = 21)** PRE+FOE + placebo	**Group III (n = 19)** SE+FOE+ nutritional supplement	**Group IV (n = 21)** SE+FOE + placebo	**Overall** ***P*-value**
	mean ± SD	mean ± SD	mean ± SD	mean ± SD	

**Right leg**					

Knee flexion	21.4 ± 6.6	21.5 ± 5.7	17.4 ± 6.4	17.1 ± 4.9	**0.04**

Knee extension	38.6 ± 10.8	39.8 ± 12.6	36.2 ± 12.2	34.9 ± 12.0	**0.01**

Hip flexion	32.3 ± 11.6	36.5 ± 11.6	32.9 ± 10.7	31.6 ± 8.7	**0.03**

Hip extension	25.8 ± 5.3	26.1 ± 8.4	22.1 ± 6.8	21.0 ± 7.9	**0.01**

**Left leg**					

Knee flexion	22.5 ± 7.0	22.0 ± 6.9	19.2 ± 6.3	19.1 ± 6.1	**0.05**

Knee extension	39.6 ± 12.7	42.8 ± 9.9	35.8 ± 13.4	36.6 ± 10.9	**0.04**

Hip flexion	34.0 ± 11.5	35.4 ± 11.0	33.8 ± 11.6	32.0 ± 9.6	NS

Hip extension	26.8 ± 6.0	25.5 ± 9.3	21.4 ± 7.3	21.9 ± 8.6	**0.01**

PRE – Progressive resistance exercises, FOE – Functionally-oriented exercises, SE – Standard exercises, SD – standard deviation, NS – statistically non-significant. Analysis of variance (ANOVA I), with the Bonferroni correction for comparison between the respective groups. Bold font highlights all statistically significant results.

**Table 4:** Muscle strength – changes in outcome measures between groups: baseline values vs. scores after 7 weeks – Multiple Range Test.

**Contrast**	**Difference**							
	**Right leg**				**Left leg**			

	Knee flexion	Knee extension	Hip flexion	Hip extension	Knee flexion	Knee extension	Hip flexion	Hip extension

**Group I vs. Group II**	0.09	-0.61	-0.28	0.55	0.44	0.38	-0.45	0.40

**Group I vs. Group III**	0.84*	0.66	1.69	1.14*	1.40*	1.48*	0.28	2.29*

**Group I vs. Group IV**	1.43*	0.98	1.06	1.98*	1.92*	1.56*	1.38	2.70*

**Group II vs. Group III**	0.74*	0.14	1.97	1.69*	0.95	2.14*	0.72	1.43*

**Group II vs. Group IV**	1.33*	3.84*	2.35*	2.38*	0.57	2.49*	1.83	1.86*

**Group III vs. Group IV**	0.05	0.32	0.37	0.68	-0.37	0.46	1.09	-0.86

* denotes a statistically significant difference.

#### Individual allocation of the Thera-Band^®^

Following the initial individual assessment of 1-RM, each subject was allocated the elastic resistive exercise band specifically calibrated (in compliance with Thera-Band^® ^colour-coding system) to suit his own muscle strength. In the present study only two Thera-Band^® ^colour codes were utilised: yellow (e.g. 50 cm of stretch of double folded band is equivalent to 20 N) and red (e.g. 50 cm of stretch of double folded band is equivalent to 30 N). The attending physiotherapist would then instruct the subject by exactly how much the double folded elastic band was to be stretched out in order to achieve the required 80% of 1-RM. The subjects' delivery was also strictly monitored throughout to minimize the risk for muscle injury.

#### Nutritional supplementation

Nutritional supplement (NUTRIDRINK^®^, strawberry flavoured, made by N.V. Nutricia – Zoetemeer, The Netherlands) was provided to the study subjects once daily, each time shortly before the commencement of their routine exercise regimen, throughout the period of 7 weeks, five times a week, with a view to establishing the impact of supplementary nutrition intake on patients undergoing specific physiotherapy programme. The nutritional supplement – a 200 ml liquid supplying 300 kcal in the form of carbohydrate (49%), lipids (35%) and protein (16%) mixture was designed to augment individual caloric intake by ca. 20%, as well as top up their recommended daily allowances of vitamins and minerals (by ca. 25%). Full (100%) compliance was assured by the trained nursing stuff (i.e. each ingestion of the nutritional supplement or placebo was strictly monitored and duly recorded in the CRFs).

#### Placebo supplementation

All subjects not receiving a nutritional supplement were given an equal volume of a substantially less nutritive (41 kcal), artificially sweetened, strawberry-flavoured liquid containing 97.5% of carbohydrates, 1% lipids and 1.5% proteins (Vitadrink^® ^manufactured by Habiron). Both the supplement and placebo were administered in unmarked containers by the nursing staff, who had no prior knowledge of their contents, nor indeed were in any way privy to the actual nature of their assignment.

### Study limitations

NUTRICIA recommends that depending on individual nutritional status and average daily energy expenditure (i.e. average level of physical activity) of the study subjects their daily nutritional supplement should range from 1 – 3 servings of NUTRIDRINK^®^, translating into 300 – 900 kcal/daily. As the present study was subject to certain budgetary constraints, the authors could not afford to offer more than 1 NUTRIDRINK^® ^per head daily; this in fact being deemed to impose a specific limitation on the study design at large.

### Statistical analyses

The statistical analysis made use of the following trait characteristics: arithmetic mean, standard deviation, standard skewness and standard kurtosis. A sample size of 22 – 23 participants per group was estimated to provide over 90% power at a significance level of *P *<*0.05*. With a view to determining the statistical significance of any differences between the first and the second assessment of the study subjects, t-Student test was applied, as well as the Paired-Sample-Comparison, with the aim of establishing the actual effectiveness of the pursued regimens.

In order to compare the results yielded by the respective study groups, one-way analysis of variance (ANOVA I) and multiple range tests (i.e. the Bonferroni method) were applied. Statistical significance was established as p < 0.05. Since individual balance and gait scores were assessed with the aid of the 0 – 2 scale (as per the modified Tinetti POMA test), a non-parametric Wilcoxon test was used for comparing the paired variables. All data were subsequently processed by STATGRAPHICS Plus v. 5.0. for Windows^® ^software package.

## Results

### Primary outcomes

Significant differences in muscle strength were noted both in favour of Group I (PRE + nutritional supplementation) and Group II (PRE + placebo), as compared to Group III (SE + nutritional supplementation) and Group IV (SE + placebo). It might therefore be reasonably inferred, despite a relatively short duration of the study, that progressive resistance exercise (PRE) regimen is clearly instrumental in appreciably improving individual muscle strength, irrespective of nutritional supplementation, at least in the volume actually offered to the present study subjects (Table 3 and 4).

With regard to individual mobility notable improvements were reported in Group III which pursued the regimen combining SE with nutritional supplementation, although, admittedly, appreciable gains in individual muscle strength (Group I) clearly did not appear to translate directly into perceptible improvement in individual mobility (Table 5 and 6).

**Table 5:** Mobility – changes in outcome measures between groups: baseline values vs. scores after 7 weeks.

**Mobility**	**Group I (n = 19)** PRE+FOE +nutritional supplement	**Group II (n = 21)** PRE+FOE + placebo	**Group III (n = 19)** SE+FOE+ nutritional supplement	**Group IV (n = 21)** SE+FOE + placebo	**Overall** ***P*-value**
	mean ± SD	mean ± SD	mean ± SD	mean ± SD	

**Tinetti – total score**	22.4 ± 5.6	22.5 ± 5.4	22.6 ± 5.2	22.1 ± 4.6	NS

**Tinetti – balance score**	12.7 ± 3.3	12.7 ± 2.7	13.1 ± 3.3	12.4 ± 2.9	**0.01**

**Tinetti – gait score**	9.7 ± 2.9	9.8 ± 2.2	9.5 ± 2.3	9.7 ± 2.4	NS

**6 MW Test (m.)**	287 ± 121	224 ± 110	299 ± 110	263 ± 105	**0.002**

PRE – Progressive resistance exercises, FOE – Functionally-oriented exercises, SE – Standard exercises, SD – standard deviation, NS – statistically non-significant. Analysis of variance (ANOVA I), with the Bonferroni correction for comparison between the respective groups. Bold font highlights all statistically significant results.

**Table 6:** Mobility – changes in outcome measures between groups: baseline values vs. scores after 7 weeks – Multiple Range Test.

**Contrast**	**Difference**			
	**Tinetti – total score**	**Tinetti balance score**	**Tinetti – gait score**	**6 MW Test (m.)**

**Group I vs. Group II**	-0.55	-0.43	-0.10	3.64

**Group I vs. Group III**	-0.42	1.32*	0.09	-4.81*

**Group I vs. Group IV**	0.76	-0.54	-0.21	2.47

**Group II vs. Group III**	0.13	1.42*	0.20	-9.93*

**Group II vs. Group IV**	1.32	0.88	-0.10	-6.29*

**Group III vs. Group IV**	1.18	-0.10	-0.31	5.11

* denotes a statistically significant difference.

### Secondary outcomes

In the groups assigned standard exercise (SE) regimen (incorporating the use of a pedal exerciser) statistically significant improvement was noted with regard to the distance covered by over 35 metres on completion of a 7-week study (Table 7), whereas in the groups following progressive resistance exercise (PRE) regimen muscle strength in both legs reached statistically significant improvement by 3.5 – 6.6 N/m over the same period (Table 8).

**Table 7:** Mobility – the within-group differences between post – and pre-intervention values

	**Group I (n = 19)** PRE+FOE+ nutritional supplement		**Group II (n = 21)** PRE+FOE +placebo		**Group III (n = 19)** SE+FOE + nutritional supplement		**Group IV (n = 21)** SE+FOE+placebo	
	mean ± SD	p value	mean ± SD	p value	mean ± SD	p value	mean ± SD	p value

Tinetti Total score	0.36 ± 2.6	0.54	-1.15 ± 3.4	0.15	0.26 ± 2.2	0.61	**1.61 ± 2.8**	**0.01**

Tinetti Balance score	0.26 ± 1.6	0.50	-0.85 ± 2.3	0.12	0.0 ± 2.2	1.0	**1.42 ± 1.9**	**0.003**

Tinetti Gait score	0.05 ± 1.2	0.85	-0.35 ± 1.6	0.34	0.1 ± 1.0	0.66	0.19 ± 2.4	0.72

6 MW Test	20.7 ± 58.8	0.14	2.35 ± 55.2	0.85	**36.4 ± 55.1**	**0.009**	**35.7 ± 58.9**	**0.01**

Use walking aids	21%		20%		5%		5%	

*p *value for test of interaction effect between the PRE and SE regimens. Bold font highlights all statistically significant results.

**Table 8:** Muscle strength – the within-group differences between post – and pre-intervention values

	**Group I (n = 19)** PRE+FOE +nutritional supplement		**Group II (n = 21)** PRE+FOE +placebo		**Group III (n = 19)** SE+FOE +nutritional supplement		**Group IV (n = 21)** SE+FOE +placebo	
	mean ± SD	p value	mean ± SD	p value	mean ± SD	p value	mean ± SD	p value

**Right leg**								

Knee flexion	3.3 ± 6.3	0.09	3.0 ± 5.4	0.06	-0.3 ± 5.8	0.65	-1.5 ± 4.3	0.79

Knee extension	**5.6 ± 9.6**	**0.02**	3.8 ± 9.7	0.08	1.7 ± 8.9	0.21	1.4 ± 9.0	0.36

Hip flexion	**5.2 ± 7.8**	**0.009**	**6.6 ± 9.7**	**0.005**	3.3 ± 11.3	0.22	3.1 ± 9.9	0.16

Hip extension	**4.6 ± 9.1**	**0.05**	**4.8 ± 6.1**	**0.003**	1.9 ± 5.8	0.31	0.7 ± 6.5	0.91

**Left leg**								

Knee flexion	**4.2 ± 5.9**	**0.02**	**4.1 ± 4.3**	**0.01**	1.5 ± 5.2	0.47	0.2 ± 3.8	0.31

Knee extension	**6.0 ± 7.9**	**0.003**	**6.0 ± 7.8**	**0.002**	2.7 ± 9.8	0.24	1.7 ± 8.7	0.17

Hip flexion	4.2 ± 12.2	0.09	**3.5 ± 8.2**	**0.02**	2.7 ± 7.8	0.14	1.5 ± 8.7	0.46

Hip extension	**4.4 ± 6.2**	**0.004**	3.1 ± 7.0	0.07	-0.2 ± 5.8	0.78	-0.2 ± 4.6	0.26

*p *value for test of interaction effect between the PRE and SE regimens. Bold font highlights all statistically significant results.

### Additional findings – body weight gains

The study subjects who followed standard exercise (SE) regimen (Group III), as well as received nutritional supplement were found to have appreciably gained in body weight by ca. 1.72 kg on average over 7 weeks (*p *= 0.01), whereas no such gains were observed in the group pursuing the progressive resistance exercise (PRE) regimen (Group I); the difference possibly attributable to the perceptibly less strenuous character of the former regimen.

## Discussion

Functional impairment in the fast-growing population of the frail elderly is generally acknowledged to be a complex issue in an urgent need of comprehensive addressing. This prompted the authors to focus primarily on seeking out such comprehensively structured interventions that would ideally combine feasibility, ease of application and overall economic viability, all with a view to their prospective wide-scale application, not least in the overstrained public health care sector [34-36].

Indeed, very few studies to date have addressed the combined effects of specifically structured exercise regimens and nutritional supplementation as an effective way of tangibly improving the strength and functionality of the frail elderly individuals, be that nursing home residents or free-living community dwellers, or indeed proposed any practical measures aimed at effectively retarding the age-induced decline in functionality before it slides down into unrecoverable dependency [3,37,38].

Since physical frailty is generally construed as a state of reduced physiological reserve associated with an increased susceptibility to disability, whereas physical inactivity and dietary inadequacies are its main contributors [39], it seemed only prudent to focus the actual investigation primarily on the following key factors as most clearly indicative of the actual nature of this complex problem: mobility, muscle strength and overall nutritional status.

Appreciable improvement of muscle strength in the lower limbs, as gained through progressive resistance training, does not directly translate into effective walking capability. As Skelton et al. observed, isolated improvements of strength and power standardised for body weight may not be sufficient to improve individual functional ability in the elderly people with already impaired functional capabilities [40]. A person may indeed be quick enough to get up from a chair and walk steadily a short distance (e.g. to reach the toilet), but would be quite challenged to venture further away without an assistive device, let alone being able to effectively pursue the activities of daily living without the necessity of contracting outside help.

Rosendahl et al. [38], even though his study population was recruited from nursing home residents (mean age 84 years; 45 min. exercise sessions pursued 5 times every fortnight over 13 weeks) with severe functional and cognitive impairment, subscribed to the view that overall improvement in physical function, as demonstrated by his study, might well hold substantial potential for being converted into higher activity level or greater self-reliance amongst the frail elderly, consequently enhancing their overall capabilities for more effective pursuit of ADL; this conclusion being fully on a par with our own findings.

Incidentally, Rosendahl did not seem to construe the individual ability to cover longer distance (as routinely assessed by the 6 MW test or other) as a significant marker of individual functional capability, opting for the assessment of the gait speed instead, which in turn seems to undermine to certain extent overall consistency of his approach, especially in view of so much praise having been lavished on the High Intensity Functional Exercise (HIFE) Programme applied throughout his study.

Considering, however, that our own exercise regimen provided for 45 min. uniform exercise sessions 5 times a week, pursued for 7 weeks (the weekend sessions being non-feasible for organisational reasons), we would be somewhat wary of accepting Rosendahl's term HIFE Programme, especially in view that each of his study subjects had his exercises individually tailored by the physiotherapist to suit his individual functional deficits. Arguably, this particular approach seems to make the actual quantification of exercise effectiveness a rather daunting task.

Besides, despite offering rather comprehensive baseline characteristics, Rosendahl neglected to offer, however, any information on whether the application of HIFE Programme actually yielded any quantifiable improvements in terms of downgrading the type of walking aids used by his subjects, which might otherwise be of some value for designing more effectively targeted exercise regimens; this particular type of study generally expected to be intrinsically focused on practical application of its findings.

The results yielded by our own study clearly support the conclusion that only standard exercises in combination with the functionally targeted ones may prove effectively instrumental in appreciably enhancing individual walking capabilities, as assessed by the 6 MW test, despite notably failing to improve overall muscle strength in the knee flexors and extensors. Admittedly, overall muscle strength in the lower limbs is just one of several key factors essentially contributing to the effective performance of independent activities, although its true significance should by no means be underestimated.

Although Bunout et al. [35] reported that overall walking capabilities in his subjects remained virtually unaffected by resistance training exercises over the 18-month span (i.e. lacked statistical significance), this might reasonably be extrapolated that ca. 50% compliance rate (to which Bunout himself freely admitted) might well be expected to yield closely similar results to 100% compliance rate obtained whilst pursuing standard exercise regimen. Also in assessing the actual walking capabilities of his subjects Bunout for some reason opted for measuring the distance covered over 12 minutes instead of applying the standard 6 MW test, which would certainly have added more credence to his findings. Another intervening factor consisted in the fact that all his subjects were involved in a 15-min. walking period prior to and after the respective resistance training sessions.

It might perhaps also be worth mentioning at this juncture that in ca. 20% of the study subjects who had been allocated progressive resistance exercise regimen a perceptible trend toward diminished reliance on assistive devices was observed (e.g. a walker was replaced with a walking cane by the end of the study), although a study embracing a much longer time span would obviously be required to determine its true, long-term statistical significance. Similar observations were also made by Fiatarone et al. [3] and Sullivan et al. [41].

The presently applied resistance exercise regimen was deliberately focused on leg extension, as both the knee and hip extensors are the muscle groups widely acknowledged to be of critical importance in executing such basic postural shifts as sit-to-stand (and vice versa) and walking, with a long-term aim of appreciably enhancing individual functional performance [42,43].

Fiatarone et al. [3], who conducted her study on older (mean age 87 years) frail nursing home residents, also reported appreciably improved overall strength in several muscle groups over a 10-week span (45 min. sessions 3 times weekly) in the subjects following intensive resistance exercise programme combined with a nutritional supplementation regimen of a closely similar type.

Bonnefoy et al. [37], whose randomised study design embraced combined nutritional supplementation and a variety of structured exercises (study population: retirement home residents; mean age over 83 years; 60 min. exercise sessions pursued 3 times weekly over 9 months), concluded that such a combined intervention strategy in the frail elderly was indeed a feasible therapeutic modality (ca. 50 – 60% success rate), even though he conspicuously failed to elucidate the actual nature of the control activity (memory), as well as his study protocol did not provide for any clear-cut discrimination in terms of the actual duration of the respective types of exercises (e.g. no juxtaposition between the progressive resistance type and the other exercises) that would reasonably merit some quantification, with a view to establishing their respective impact on the outcomes under study.

The study pursued by Bunout et al. [35], embracing the free-living 70-year olds, was altogether different in its design, though, as it spanned 18 months worth of follow-up during which his subjects were assessed four times, although compliance rates with respect to both their attendance at the resistance training sessions (1 h sessions twice weekly) and nutritional supplementation were relatively low (56% and 48%, respectively), giving therefore some grounds to doubt the actual effectiveness of both regimens.

Since the composition of the nutritional supplement used by Bunout was on a par with the one offered to our own study subjects (NUTRIDRINK^®^), overall effectiveness of the applied supplementation regimen should be assessed in terms of the respective compliance rates. The fact that Bunout had his nutritional supplement diluted in a soup or porridge seems to have significantly contributed to a relatively low compliance rate.

The authors fully appreciate the concerns voiced by Paul L. de Vreede [44], though, who pointed out that muscle strength gain induced by resistance exercise regimen is invariably lost after a relatively short detraining period (i.e. when a regular pursuit of resistance exercise regimen is discontinued), whereupon the body readily adjusts to the diminished physiological demand and consequently all the beneficial adaptations achieved throughout the exercise period may in fact be lost.

Those vital concerns actually prompted the authors to discuss the issue of detraining in some detail with the study subjects, ostensibly in an attempt to encourage them to continue the regimens on their own accord, once the study has been concluded. As it happened, most of them actually proved quite enthusiastic about performing the various exercises and seemed to understand well enough that a consistent and diligent pursuit of the prescribed regimens well beyond the actual time frame of the study was actually supposed to help them sustain the already restored capabilities, which they unequivocally regarded as a tangible, long-term benefit. Even though the present study design did not expressly provide for any follow-up and no data are therefore available to substantiate the authors' claim, they do have good reasons to believe that with regard to a number of former study subjects the obvious benefits of continuation proved a persuasive enough argument.

In his study De Vreede demonstrated the superiority of functional-task exercises over the resistance type in terms of the results of the former standing to be preserved for much longer. His findings are in fact very much on a par with the results of our own investigation, where the multi-sensory exercises, the core component of FOE, were found to demonstrate by far the greatest potential with regard to enhancing individual functional capabilities, as they effectively stimulate individual balance, overall movement co-ordination and reaction time in responding to situations of impending postural risk, e.g. incidental fall, as well as prepare a person much better to cope effectively afterwards. Skelton et al. seems to share this view and even ventures to say that enhanced individual functional capabilities specifically contribute to tangible improvement in overall quality of life for older people [45].

Although the therapeutic value of SE should on no account be underestimated, as they effectively prevent muscle cramps, enhance the range of joint movement and generally improve peripheral circulation, this particular type of exercises may never suffice as a self-contained therapeutic option in addressing the complex issue of impaired functional capabilities.

Even though the authors strongly believe that for best therapeutic results both regimens (SE + FOE) should always be pursued in combination, it is actually the FOE type that can safely be recommended to a much more diversified population of frail elderly (inclusive of those suffering from a diversity of neurological and cardio-vascular disorders, as well as those remaining in post-operative recuperation), irrespective of their specific social setting, as they have positive impact over a significantly larger number of bodily systems and functions.

In seeking to design optimum exercise interventions programme de Vreede advocated that specifically targeted, functional-task exercises be primarily considered in view of their substantial potential for long-term effectiveness. The authors fully support this view in so far as more in-depth research is still required in order to determine beyond reasonable doubt which specific exercise interventions of the FOE type hold by far the greatest potential to yield the most promising, long-term results and should therefore primarily be considered as the core element of any viable physical rehabilitation programme under development; overall validity and persuasive character of de Vreede's findings notwithstanding [44].

Admittedly, overall complexity of the issue addressed by the present study clearly merits pursuit of further studies of a closely similar, multi-factorial intervention design, though preferably conducted over much longer time span, as well as best targeting the over 60s as the population standing by far the best chance of a successful outcome, in order to verify whether the present findings, clearly encouraging as they appear, might actually have sufficient potential to develop into a trend of indisputable clinical significance, especially in terms of possible application in a comprehensively designed, nationwide programme specifically aimed at addressing the problem of a steadily growing proportion of the frail seniors dependent for their activities of daily living.

## Conclusion

Comprehensively structured, high-intensity regimen made up of diverse exercise types, i.e. functionally-oriented, progressive resistance and standard ones, preferably if combined with nutritional supplementation offered in adequate volume, is believed to demonstrate clear potential for appreciably improving overall functional status in the frail elderly, especially in terms of individual walking capacity and muscle strength, as well as for possible downgrading of any currently used assistive devices. It would also appear that consistent application of functionally-oriented exercise regimens making active use of simple, easy-to-use props may significantly enhance individual balance.

## Abbreviations

PRE: Progressive resistance exercises; FOE: Functionally-oriented exercises; SE: Standard exercises; 6 MW: Six-Minute Walk test; Tinetti's POMA test: Tinetti's Performance Oriented Mobility Assessment test; BMI: Body Mass Index; BBS: Berg Balance Scale test; MMSE: Mini-Mental State Examination; ADL: Activities of Daily Living; IADL: Instrumental Activities of Daily Living; MNA: Mini Nutritional Assessment; CRF: Case Report Form(s); GCP: Good Clinical Practice guidelines; SD: standard deviation; LDL: Low-density lipoprotein cholesterol; HDL: High-density lipoprotein cholesterol; 1 RM: One repetition maximum; HIFE: High Intensity Functional Exercise.

## Competing interests

The authors declare that they have no competing interests.

## Authors' contributions

MZ contributed to overall design of the study, inclusive of the intervention set-up, contributed to analyses and interpretation of results and drafted the manuscript. CHS made key contribution to the actual conception and overall design of the study, supervised all its aspects throughout and critically revised the final draft of the manuscript. TG facilitated the actual implementation of the study regimens, generally managed the project and contributed to the preparation of the manuscript in terms of overall analytical consistency.

All authors read and approved the final manuscript.

## Pre-publication history

The pre-publication history for this paper can be accessed here:


